# The effect of gestational age, low birth weight and parity on birth asphyxia among neonates in sub-Saharan Africa: systematic review and meta-analysis: 2021

**DOI:** 10.1186/s13052-022-01307-5

**Published:** 2022-07-15

**Authors:** Masresha Asmare Techane, Tewodros Getaneh Alemu, Chalachew Adugna Wubneh, Getaneh Mulualem Belay, Tadesse Tarik Tamir, Addis Bilal Muhye, Destaye Guadie Kassie, Amare Wondim, Bewuketu Terefe, Bethelihem Tigabu Tarekegn, Mohammed Seid Ali, Beletech Fentie, Almaz Tefera Gonete, Berhan Tekeba, Selam Fisiha Kassa, Bogale Kassahun Desta, Amare Demsie Ayele, Melkamu Tilahun Dessie, Kendalem Asmare Atalell, Nega Tezera Assimamaw

**Affiliations:** 1 Department of Pediatric and Child Health Nursing, School of Nursing, College of Medicine and Health Sciences, University of Gondar, Gondar, Ethiopia; 2 Department of Community health Nursing, School of Nursing, College of Medicine and health science, University of Gondar, Gondar, Ethiopia

**Keywords:** Neonate, Newborn, Birth asphyxia, Sub-Saharan Africa

## Abstract

**Background:**

Despite simple and proven cost-effective measures were available to prevent birth asphyxia; studies suggested that there has been limited progress in preventing birth asphyxia even in healthy full-term neonates. In Sub-Saharan Africa, Inconsistency of magnitude of birth asphyxia and its association gestational age, Low birth Weight and Parity among different studies has been observed through time.

**Objective:**

This study aimed to estimate the Pooled magnitude of birth asphyxia and its association with gestational age, Low birth Weight and Parity among Neonates in Sub-Saharan Africa.

**Method:**

PubMed, Cochrane library and Google scholar databases were searched for relevant literatures. In addition, reference lists of included studies were retrieved to obtain birth asphyxia related articles. Appropriate search term was established and used to retrieve studies from databases. Searching was limited to cohort, cross-sectional, and case-control studies conducted in Sub-Saharan africa and published in English language. Joanna Briggs Institute Meta-Analysis of Statistics Assessment and Review Instrument (JBI-MAStARI) was used for critical appraisal of studies. Heterogeneity across the included studies was evaluated by using the inconsistency index (I^2^) test. Funnel plot and the Egger’s regression test were used to test publication bias. A weighted inverse variance random effects- model was used to estimate the pooled prevalence of birth asphyxia among neonates in Sub-Saharan Africa. STATA™ version 11softwarewasused to conduct the meta-analysis.

**Result:**

A total of 40 studies with 176,334 study participants were included in this systematic review and meta-analysis. The overall pooled magnitude of birth asphyxia in Sub-Saharan Africa was 17.28% (95% CI; (15.5, 19.04). low birth weight (AOR = 2.58(95% CI: 1.36, 4.88)), primigravida (AOR = 1.15 (95% CI: 0.84, 1.46) andMeconium-stained amniotic fluid (AOR = 6(95% CI: 3.69, 9.74)) werevariables significantly associated with the pooled prevalence of birth asphyxia.

**Conclusion:**

The pooled magnitude of birth asphyxia was found to be high in Sub-Saharan Africa. Low birthweight and Meconium-stained amniotic fluid were variables significantly associated with birth asphyxia in Sub-Saharan Africa. Hence, it is better to develop early detection and management strategies for the affected neonates with low birth weight and born from mothers intrapartum meconium stained amniotic fluid.

## Introduction

Birth asphyxia can be defined as the inability to initiate and sustainedbreathing at birth [1]. Asphyxia is a lack of blood flow or gas exchange which could occur immediately before, during, or after the birth process. Causes of asphyxia include prenatal or immediate post-natal compromise of gas exchange resulting in lack of oxygen to the vital organs with subsequent hypoxemia and hypercapnia. If the hypoxemia is severe enough, vital organs will develop an oxygen debt, anaerobic glycolysis and lactic acidosis.

It is a situation that arises when there is impairment of blood gas exchange, which leads to hypoxemia, hypercapnia, metabolic acidosis, and multi organ failure [2]. According to the International Classification of Diseases, Tenth Revision (ICD-10) of the World Health Organization (WHO), birth asphyxia can be defined and classified by using the APGAR score at 1 and 5 minutes as mild, moderate, and severe [3].

The global incidence of birth asphyxia is estimated at 2 to 10 per 1000 among term newborns [4] and it is higher in developing countries than in developed countries as a result of the reduced availability of skilled care provided during delivery. Globally, birth asphyxia accounts for more than 24% of neonatal mortality [5]. Birth asphyxia is one of the leading causes of neonatal mortality in low and middle-income countries and also the main cause of long-term illnesses including mental retardation, cerebral palsy, and other neurodevelopment disorders [6]. In Africa, birth asphyxia accounts 24.0%, of which two-third of the incidence (15.9%)occurred in East and Central Africa [7].

Causes of birth Asphyxia may be a maternal or fetal condition that happens before birth, during birth, or a combination of these [8–11]. In different studies, many determinant factors of birth asphyxia have been detected, but the reduction of cerebral blood flow by any mechanism is the exact cause of birth asphyxia [12]. Risk factors of birth asphyxia that occur before birthincludes severe maternal hypotension or hypertensive diseases during pregnancy [12–14], antepartum hemorrhage [15–17],less antenatal care visits,oligohydramnios,young maternal age,advanced maternal age, and low educational status [10, 18–23]. During birth, birth asphyxia can be associated with prolonged,home delivery,obstructed labor, oxytocin use,malpresentation,andmeconium-stained amniotic fluid [9, 11, 18–21, 24–28]. Fetal risk factors associated with birth asphyxia include low birth weight,multiple gestation, tight nuchal cord, preterm delivery, and fetal distress [9, 11, 18, 19, 21, 25–28].

Birth asphyxia leads to various outcomes in the life of the neonate, such as multi-organ dysfunction,death, severe neurodevelopmentdelay, motor delay, cerebral delay, and hypoxic ischemic encephalopathy (HIE) [29–31].

In Sub-Saharan Africathe burden of birth asphyxia is critical and public health problem that happened as a result of inadequate obstetrics health coverage, inaccessible health facilities, sociocultural norms, poor educational levels, shortage in health workers and supplies and poor health care spending. Likewise, facility deliveries, skilled delivery assistance and adequate antenatal visitswas lower in Sub-Saharan Africa regions [21, 29, 32, 33].

Despite simple and proven cost-effective measures were available to prevent birth asphyxia, studies suggested that there has been limited progress in preventing birth asphyxia even in healthy full-term neonates [3].

As far as our search, the pooled prevalence of birth asphyxia was not previously investigated in sub-Saharan Africa. The findings of previous studies on the magnitude of birth asphyxia were inconsistent and ranged from 3.1% [24] to 39.7% [27] across the sub-Saharan African countries. Hence, this systematic review and meta-analysis study was aimed at determining the pooled estimate prevalence of birth asphyxia and its association with gestational age, low birth weight, and parity among neonates in Sub-Saharan Africa.

## Method and materials

### Searching strategy and eligibility criteria

The Preferred Reporting Items for Systematic Review and Meta-Analysis statement (PRISMA) guideline [34] was used to report the results of this systematic review and meta-analysis.and,it is registered in the Prospero database as (PROSPERO 2021: CRD42021288351).

In order to obtain the significant articles international electronics databases such as PubMed, Google Scholar, and Cochrane library were retrieved. Two independent authors were assigned in order to systematically searching articles.

In addition, other significant articles were retrieved manually from the gray literature by cross-referencing. The core search terms and phrases were “newborn”, “neonate”, “birth asphyxia”, “perinatal asphyxia”, “magnitude of birth asphyxia”, “and associated factors”, “Ethiopia”. The search strategies were developed using different Boolean operators. Particularly, to fit the advanced PubMed database, the following search strategy was applied: [(newborn [MeSH Terms] OR neonate OR newborn baby AND (birth asphyxia [MeSH Terms] OR perinatal asphyxia) AND prevalence [MeSH Terms] OR incidence OR burden OR magnitude OR epidemiology AND (Associated factors) OR predictors OR determinant factors OR risk factors OR predisposing factors OR factors AND (“sub-Saharan Afric.

Studies that reported the prevalence and/ or associated factor of birth asphyxia using analytical cross-sectional, cohort, and case-control studies and published in English before October 28, 2021 were included. On the other hand, articles without an abstract and/ or full-text, studies that failed to determine the anticipated outcome of interest, and those studies with qualitative study design were excluded.

### Study variables and study selection process

In this systematic review and meta-analysis, associated factors (primigravida, low birth weight and preterm gestational age) that increase the occurrence of birth asphyxia were considered as exposure variables to estimate their effects on the magnitude of birth asphyxiaand the magnitude of birth asphyxia was considered as the outcome variable of this study.

### Study selection process, methods of data extraction and quality assessment

In order to remove duplicated studies, the retrieved articles were exported to reference manager software, Endnoteversion7. Two authors (MasreshaAsmareTechane (MAT) and SelamFisihaKassa (SFK)) screened and assessed the titles and abstracts of studies, followed by full-text assessments independently and systematically. Disagreements were resolved by consensus and discussion with other authors.

Data were extracted by using the standardized Microsoft Excel data extraction form. Name of the first author, year of publication, country, region, study design, sample size, number of outcomes, prevalence (magnitude), risk estimate (Odds Ratio, RR) with 95% confidence interval (CI) and associated factors were extracted from the included articles. The quality of the included studies was evaluated by using The Joanna Briggs Institute (JBI) quality appraisal checklist [35]. Studies were considered for meta-analysis and categorized as low risk for poor quality when it scored 50% and above of the quality assessment indicators (Table 1).

**Table 1:** Characteristics and Quality Status of the Studies Included to Assess the Pooled Magnitude of birth asphyxia in Sub-Saharan Africa

ID	first author	Yearof publication	Country	Region	Study design	Sample size	Prevalence%	Quality
1	Uwingabire.Fetal	2019	Rwanda	East Africa	Cross-sectional	340	39.70	Low risk
2	Abdo et al	2019	Ethiopia	East Africa	Cross-sectional	279	15.10	Low risk
3	G/her GT et al	2020	Ethiopia	East Africa	Cross-sectional	282	18.00	Low risk
4	Gebreheat G et al	2018	Ethiopia	East Africa	Cross-sectional	422	22.10	Low risk
5	Berhe YZ et al	2020	Ethiopia	East Africa	case-control	390	–	Low risk
6	Tasew H et al	2018	Ethiopia	East Africa	case-control	264	–	Low risk
7	Gebreslasie K et al	2020	Ethiopia	East Africa	Cross-sectional	648	12.70	Low risk
8	Jamie AH et al	2019	Ethiopia	East Africa	Cross-sectional	258	31.60	Low risk
9	Ibrahim A et al	2017	Ethiopia	East Africa	Cross-sectional	9736	3.10	Low risk
10	Wayessa ZJ et al	2018	Ethiopia	East Africa	Cross-sectional	371	12.50	Low risk
11	Getachew B et al	2020	Ethiopia	East Africa	Cross-sectional	352	11.50	Low risk
12	Alemu A et al	2019	Ethiopia	East Africa	Cross-sectional	262	32.8	Low risk
13	Mamo SA et al	2020	Ethiopia	East Africa	Cross-sectional	311	41.20	Low risk
14	Ayele MW et al	2019	Ethiopia	East Africa	case-control	429	–	Low risk
15	Gudayu TW et al	2017	Ethiopia	East Africa	Cross-sectional	261	13.80	Low risk
16	Wosenu L et al	2018	Ethiopia	East Africa	case-control	273	–	Low risk
17	Woday A et al	2019	Ethiopia	East Africa	Cross-sectional	345	22.6	Low risk
18	Meshesha ADetal	2020	Ethiopia	East Africa	case-control	386	–	Low risk
19	Demisse AG et al	2017	Ethiopia	East Africa	Cross-sectional	769	12.5	Low risk
20	Kibret Y et a	2018	Ethiopia	East Africa	case-control	380		Low risk
21	Mulugeta T et al	2020	Ethiopia	East Africa	case-control	213		Low risk
22	Selamu A et al	2019	Ethiopia	East Africa	Cross-sectional	371	20	Low risk
23	G/medhin M et al	2020	Ethiopia	East Africa	case-control	662		Low risk
24	Asfere NW et al	2018	Ethiopia	East Africa	Cross-sectional	154	29.9	Low risk
25	Bayih WA et al	2020	Ethiopia	East Africa	Cross-sectional	582	28.4	Low risk
26	Lake EA et al	2019	Ethiopia	East Africa	Cross-sectional	278	25.7	Low risk
27	Gebregziabher GT etal	2020	Ethiopia	East Africa	Cross-sectional	267	18	Low risk
28	Onyriuak et al	2006	Nigeria	West Africa	Cross-sectional	2208	8.38	Low risk
29	IgeOO et al	2011	Nigeria	West Africa	Cross-sectional	398	12.6	Low risk
30	G. I. McgilUgwu et al	2012	Nigeria	West Africa	retrospective chohort	26,000	3.3	Low risk
31	Halloran DR et al	2008	Zambia	East Africa	Cross-sectional	182	23	Low risk
32	Sepeku A et al	2011	Tanzania	East Africa	Cross-sectional	192	21.1	Low risk
33	Kibai K et al	2017	Kenya	East Africa	Cross-sectional	422	29.1	Low risk
34	Gichogo M et al	2018	Kenya	East Africa	Cross-sectional	237	5.1	Low risk
35	Abkika BM et al	2018	Chad	Central Africa	Cross-sectional	7254	5.1	Low risk
36	Biselele T et al	2013	Democratic Republic Congo	Central Africa	Cross-sectional	902	4.4	Low risk
37	Mande et al	2018	Democratic Republic Congo	Central Africa	Cross-sectional	612	19.4	Low risk
38	Foumane P et al	2013	Cameron	West Africa	case-control	117	–	Low risk
39	K. J. Nathoo et al.	1990	Zimbabwe	East Africa	case-control	225	–	Low risk
40	Iran J Child Neuroletal	2013	Cameron	West Africa	case-control	1117	8.05	Low risk

### Data processing and analysis

The data were extracted from Microsoft Excel and analyzed using STATA Version 11. Meta-analysis was performed using statistical software. The funnel plot was used to check for publication bias, and Egger’s regression test was used to check for it more objectively [36]. Heterogeneity of studies was quantified using the I-squared statistic, in which 25, 50, and 75% represented low, moderate, and high heterogeneity, respectively [37, 38]. Given that we found significant heterogeneity among the studies (I^2^ = 98.4%), Pooled analysis was conducted by using a weighted inverse variance random-effects model [39]. A sensitivity analysis was employed to see the effect of a single study on the overall estimation. For the second outcome, the odds ratio and relative risk were used to ascertain the association between determinant factors and outcome variables in the included articles.

### Operational definition

Meconium-staned amniotic fluid: the presence of meconium in the amniotic fluid which changes the color of the liquor from clear to various shades of green, yellow or brownish color depending on the degree of meconium stained liquor [40].

## Result

### Searching results, characteristics and quality of the included studies

**Fig. 1:**
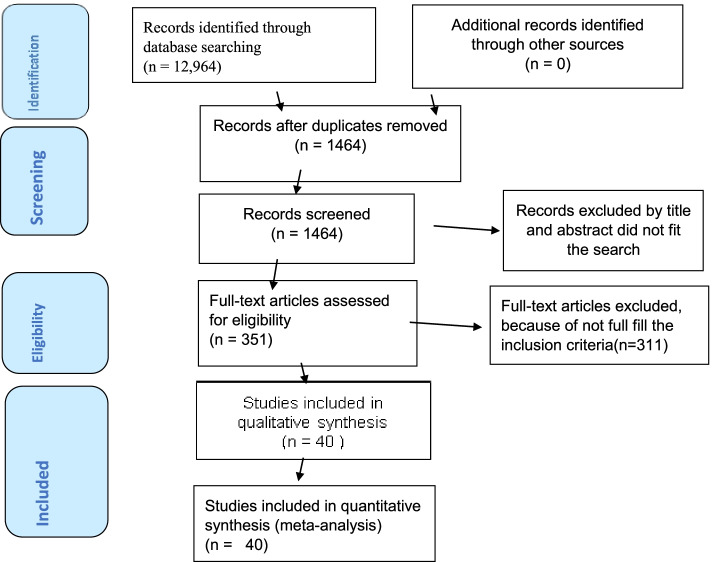
A PRISMA flow diagram of articles screening and process of selection

The search strategy retrieved 449 from Pub Med, 15 from Cochrane library and 12,500 from Google Scholar. 11,500 articles were removed due to duplicates, 657 due to unmatched title and abstracts, 456 due to study area. Three hundred fifty-one (351) articles were selected for the full text review. After full text reviews, 311 articlesdidn’t report the outcome of interest and excluded from the analysis. Finally, forty [40] articles were included in this systematic review and meta-analysis to estimate the magnitude of birth asphyxia and its association with parity, low birth weight and preterm gestational age in Sub-Saharan Africa (Fig. 1).

Thirty-two studies were found in East Africa [10, 11, 13, 15–19, 21, 22, 24, 27, 28, 41], Five in west Africa [8, 25, 26, 42, 43] and three in central Africa [44]. Most of the studies were conducted using a cross-sectional study design. In terms of publication year, nine studies were published prior to 2017, and 31 studies were published between January 2017 and December 2021(Table 1).

The JBI quality appraisal criteria established for cross-sectional, cohort and case-control study design were used to appraise the included studies. The studies included in this systematic review and meta-analysis had no considerable risk. Therefore, all the studies were considered.

### Magnitude of birth asphyxia

**Fig. 2:**
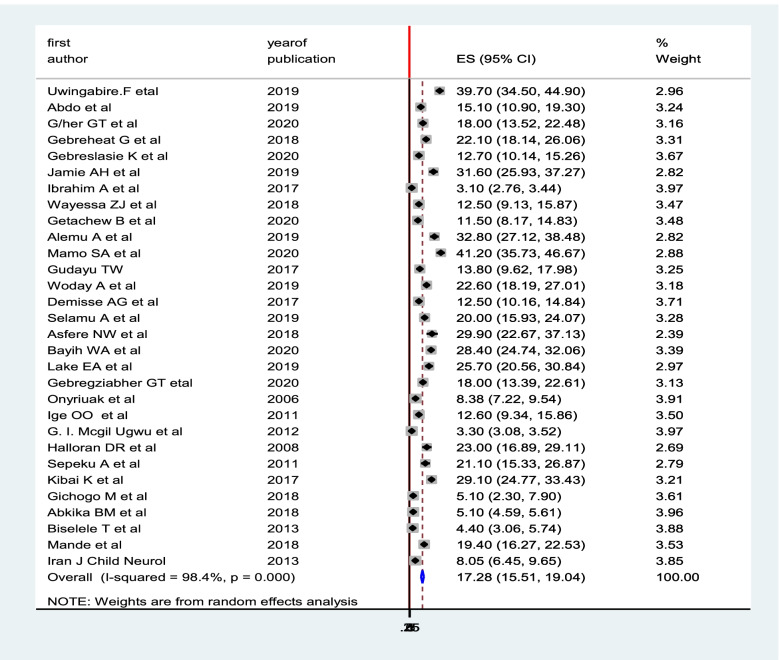
The pooled prevalence of birth asphyxia among neonates in sub-Saharan Africa

A total of 40 studies with 176,334 participants were analyzed in the meta-analysis to estimate the pooled prevalence of birth asphyxia in Sub-Saharan Africa. Consequently, the overall pooled prevalence of birth asphyxia was17.28% (95% CI; (15.5,19.04); I^2^ = 98.4% (Fig. 2).

### Subgroup analysis, publication bias and sensitivity analysis

**Fig. 3:**
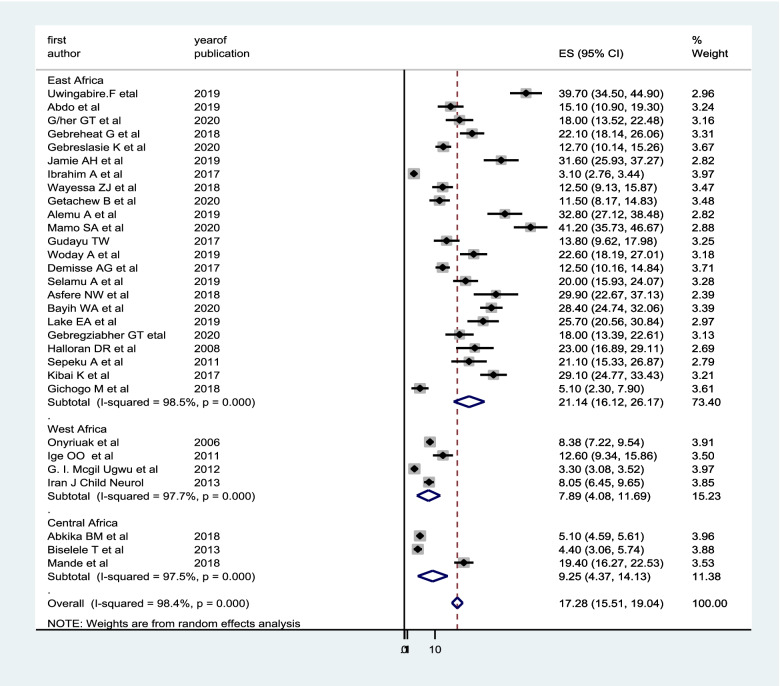
Sub group analysis of magnitude of birth asphyxia by region in Sub-Saharan Africa

We have done subgroup analysis by using region and study design of the included studies. Our subgroup analysis based on regions the study showed that the highest pooled prevalence of birth asphyxia was observed from studies done in East Africa (21.14%; 95% CI: 16.12, 26.17) (Fig. 3). But no any difference in the magnitude of birth asphyxia with study design.

**Fig. 4:**
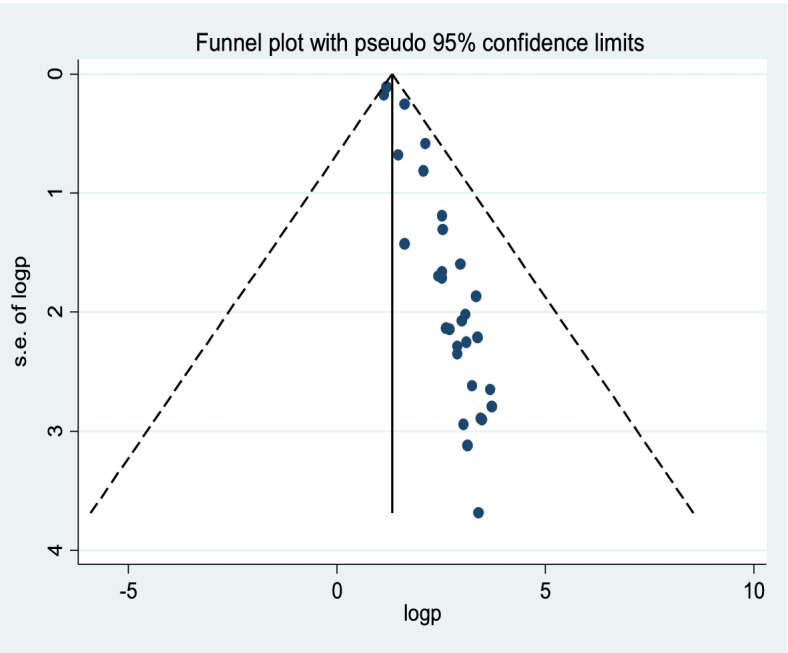
Funnel plot to determine publication bias among the included studies

Publication bias was evaluated by a funnel plot and the Egger’s regression test. A funnel plot showed asymmetrical distribution (Fig. 4) subjectively indicates the presence of publication bias. In addition, objectively the Egger’s regression test *p*-value was 0.000, which indicated the presence of publication bias.

**fig. 5:**
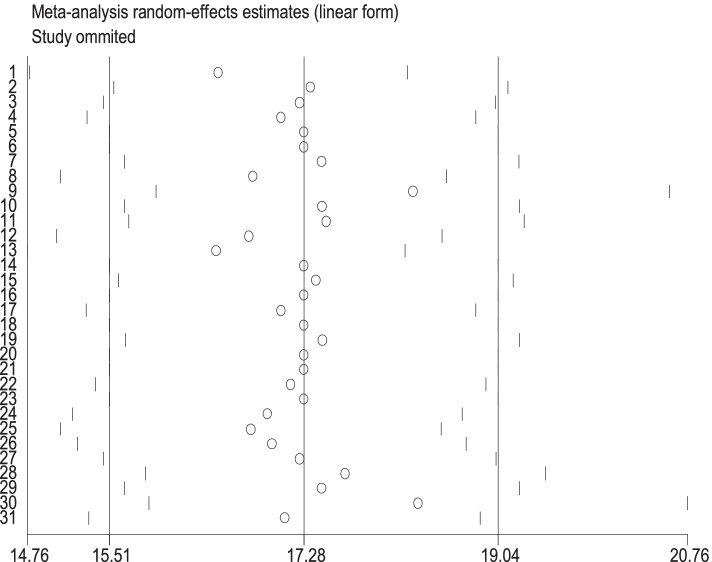
Sensitivity analysis of the included studies

We have conducted a sensitivity analysis to assess the weight of every study on the pooled effect size of the magnitude of birth asphyxia. The sensitivity analysis using the Der Simonian-Laird random-effects model showed that there was no single study that affected the overall magnitude of birth asphyxia in Sub-Saharan Africa (Fig. 5).

### Associated factors of birth asphyxia

**Fig. 6:**
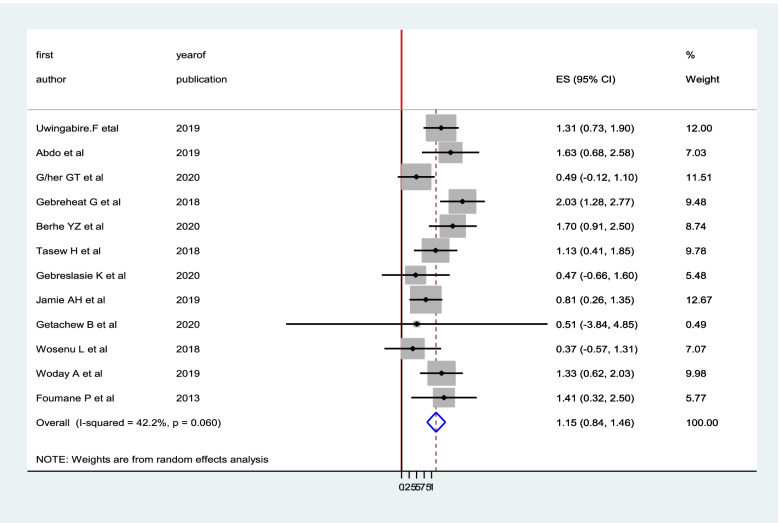
the pooled effect of parity on birth asphyxia in Sub-Saharan Africa

From the included forty studies, twelve [10, 11, 13, 15–19, 24, 27, 28, 33] studies reported the association between parity and birth asphyxia. The pooled adjusted odds ratio from these studies was 1.15 (95% CI: 0.84, 1.46), showing that the odds of birth asphyxia were 1.15 higher in neonates born from primigravida mothers than their counter parts, and it is not statistically significant (Fig. 6).

**Fig. 7:**
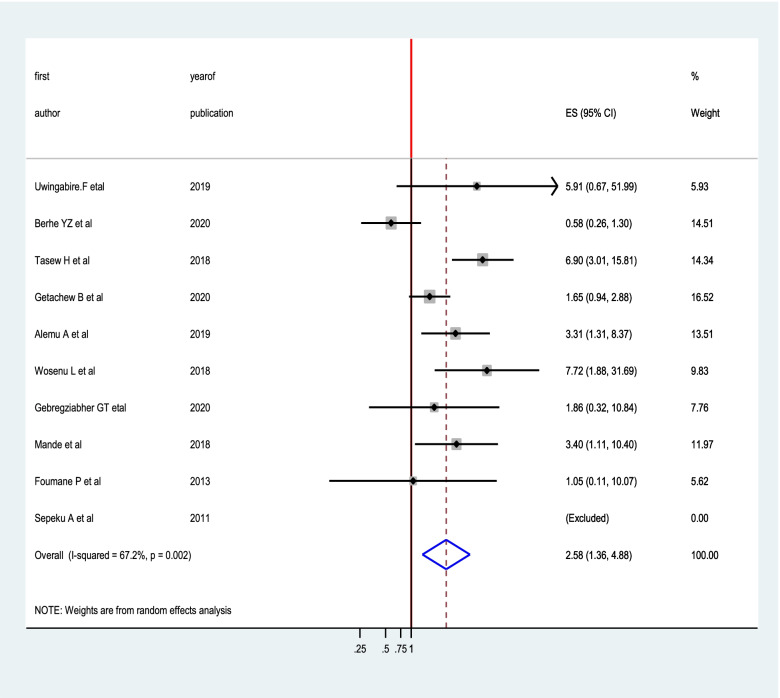
the pooled effect of low birth weight on birth asphyxia in sub-Saharan Africa

Out of the included forty studies, the association between low birth weight and birth asphyxia was reported in eight studies [10, 11, 15–17, 19, 27, 44]. The pooled oddsratio was 2.58(95% CI: 1.36, 4.88), suggesting that the risk of developing birth asphyxiawas 2.58times higher among newborns with low birth weight as compared to newborns with normal birth weight (Fig. 7).

**Fig. 8:**
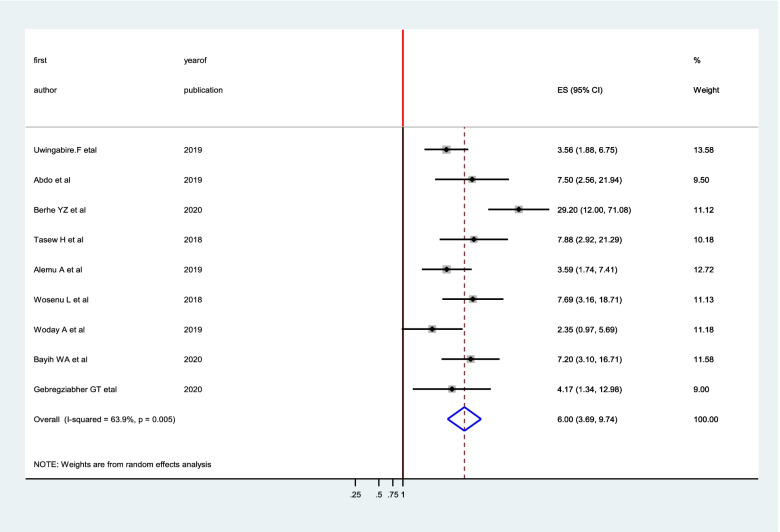
The pooled effect of meconium-stained amniotic fluid on birth asphyxia in Sub-Saharan Africa

From the included forty studies, the association between meconium stained amniotic fluid and birth asphyxia was reported in nine studies [10, 11, 13, 16, 18, 19, 28, 44]. The pooled oddsratio was 6 (95% CI: 3.69, 9.74), suggesting that the risk of developing birth asphyxiawas 6 times higher among newborns with meconium stained amniotic fluid as compared to newborns with clear amniotic fluid (Fig. 8).

**Fig. 9:**
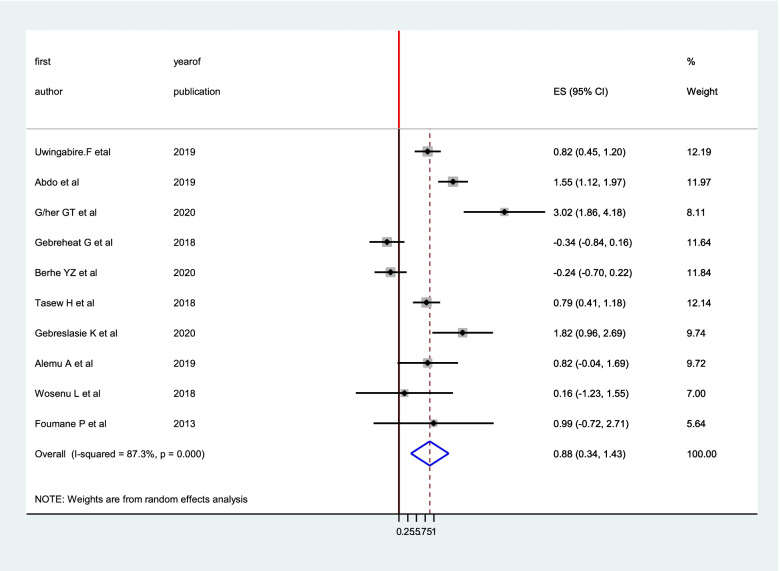
The pooled effect of gestational age on birth asphyxia in Sub-Saharan Africa

Out of the included forty studies, the association between gestational age and birth asphyxia was reported in ten studies [10, 11, 13, 15, 16, 18, 27]. The pooled odds ratio was 0.88 (95% CI: 0.34, 1.43), suggesting that the risk of developing birth asphyxia was 22% among preterm newborns as compared to newborns with term gestational age (Fig. 9).

## Discussion

In developing countries, birth asphyxia remains the main cause of neonatal morbidity and mortality [25, 30, 42, 45, 46]. As far as our exhaustive searching, there are no previous systematic reviews and meta-analyses done to estimate the pooled prevalence of birth asphyxia in Sub-Saharan Africa. As findings from various studies showed that the magnitude of birth asphyxia is variable and its association with parity, gestational age, meconium stained amniotic fluid, and low birth weight were reported inconsistentlyand not well investigated [16, 18, 19, 27, 28]. As a result, this study was aimed to estimate the pooled prevalence of birth asphyxia and its association with Parity, gestational age, meconium-stained amniotic fluid and low birth weight in Sub-Saharan Africa.

In our study, the pooled prevalence of birth asphyxia in Sub-Saharan Africa was found tobe17.28% (95% CI; (15.5,19.04). This finding is consistent with findings from other systematic review and meta-analysis done in Central and West Africa 15.9% [7]. However, our study finding was higher than studies conducted in South Africa 2.6% [47]. This variation might be due to high level of facility deliveries, skilled delivery assistance, antenatal visits and appropriate implementations of neonatal resuscitation programme in South Africa as compared to in Sub-Saharan Africa [48]. On the other hand, the findings of this study were lower than those found in other systematic reviews and meta-analysis conducted in Ethiopia, at 19.3% [49]. The possible explanation for this discrepancy may be due to the variation in study setting, study design, study population, and level of awareness with regard to poor birth outcomes for the general population, in community engagement in Ethiopia’s maternal health issues, and the differences in the implementation of services for mothers and their new-born babies as compared with participants from other Sub-Saharan African countries.

The magnitude of birth asphyxia varied greatly in the included studies, ranging from 3.1% [24] to 39.7% [27]. However, our subgroup analysis based on study location showed that the highest pooled prevalence was observed from studies done in East Africa (41.4%; 95% CI: 33.9, 48.8). A possible explanation for this variation could be the differences in healthcare facilities; With emerging an inexpensive technology, the developed nations prevention and treatment of birth asphyxia can more feasibly reach those at risk as compare to resource-limiting settings. Additionally, developed nations may have a better screening strategy of postnatal asphyxia and management of idiopathic etiologies which may help to reach both a near eradication of mortality related with birth asphyxia and reduces in its impairment.

This finding reveals the presence of a strong association between birth asphyxia and low birth weight. The odds of a newborn developing birth asphyxia was 2.58 times higher among newborns with low birth weight than among newborns with normal birth weight. This finding is in line with various studies conducted in Indonesia [50], Pakistan [51], Nigeria [25], Zambia [8], and Ethiopia [49]. This might be due to the fact that a newborn with low birth weight has poor lung surfactant, with immature lungs and weak respiratory muscles and curved ribs, which results in birth asphyxia [52, 53].

This systematic review and meta-analysis alsoshowed that the presence of meconium-stained amniotic fluid increases the occurrence of birth asphyxia. This finding is consistent with studies conducted in India [52], Pakistan [51], Indonesia [50] and Ethiopia [11, 13, 17, 19, 49]. This may be due to the fact that meconium containing amniotic fluid increases the occurrence of meconium aspiration during intrauterine gasping or during the initial breaths taken after birth, which may cause acute airway obstruction, surfactant dysfunction or inactivation [54, 55].

### Limitation

This study had its limitations. Primarily, most of the studies included for this analysis had a small sample size, which could have a significant effect on the estimated pooled prevalence of birth asphyxia. Furthermore, majority of studies included in this systematic review and meta-analysis were conducted in East Africa, which may be an underrepresentation for the other region of sub-Saharan Africa. Since it is a first systematic review, lack of enough literature and use odds ratio to estimate the predictor variables may be affected by other confounding variables. Moreover, only articles and reports published in English were considered in this review, which sought to investigate birth asphyxia in the Sub-Saharan Africa. In addition, the majority of studies included in the review were cross-sectional in nature, which limited our ability to assess cause–effect relationships and might have resulted in the outcome variable being affected by other confounding variables.

## Conclusion

Findings from this study indicated that birth asphyxia in Sub-Saharan was Still the major public health problem. This study also noted that birth asphyxia was significantly associated with low birth weight and meconium-stained amniotic fluid. Hence, it is better to assess all neonates with birth asphyxia for low birth weight and intrapartum meconium-stained amniotic fluid. Moreover, further research is needed to identify other predictors of birth asphyxia in Sub-Saharan Africa.

## Data Availability

All data generated or analyzed during the current systematic review and meta-analysis is available from the corresponding author upon reasonable request.

## Abbreviations

AOR: Adjusted Odds Ratio
CI: Confidence Interval,HIE:hypoxic ischemic encephalopathy, ICD-10:International Classification of Dis eases
Tenth Revision,JBI: Joanna Briggs Institute
OR: Odds Ratio
PRISMA: Preferred Reporting Items for Systematic Reviews and Meta-Analyses
WHO: World Health Organization
